# Blood-brain barrier dysfunction in cerebral arteriovenous malformations. A murine model of hypoperfusion-reperfusion injury assessed with dynamic contrast-enhanced MRI

**DOI:** 10.1016/j.bas.2025.105871

**Published:** 2025-11-10

**Authors:** Leire Pedrosa, Alejandra Mosteiro, Marta Codes, Gloria Cabrera, Laura Aguado, Anna M. Planas, Sergio Amaro, Abraham Martin, Ana Rodríguez-Hernández, Ramon Torné

**Affiliations:** a Instituto de Investigaciones Biomédicas August Pi i Sunyer (IDIBAPS), Barcelona, Spain; b University of Barcelona, Barcelona, Spain; c Department of Neurosurgery, Hospital Clinic of Barcelona, Barcelona, Spain; d Achucarro Basque Center for Neuroscience, Leioa, Spain; e Center for Cooperative Research in Biomaterials (CIC biomaGUNE), Basque Research and Technology Alliance (BRTA), Donostia–San Sebastián, Spain; f Department of Neuroscience and Experimental Therapeutics, Institute for Biomedical Research of Barcelona (IIBB), Spanish Research Council (CSIC), 08036, Barcelona, Spain; g Comprehensive Stroke Unit, Neurology, Hospital Clinic of Barcelona, Barcelona, Spain; h Ikerbasque Basque Foundation for Science, Bilbao, Spain; i Department of Neurosurgery, Germans Trias i Pujol University Hospital, Barcelona, Spain; j Department of Interventional Neuroradiology, Hospital Clínic of Barcelona, Barcelona, Spain

**Keywords:** Blood-brain barrier, Brain arteriovenous malformation, DCE-MRI, Reperfusion, Permeability, Rat model

## Abstract

**Introduction:**

The presence of an arteriovenous malformation in the brain (bAVM) induces hemodynamic changes, namely chronic hypoperfusion, loss of autoregulation and venous congestion, leading to blood-brain barrier (BBB) dysfunction. Conversely, bAVM treatment is thought to modify this longstanding hemodynamic condition abruptly, potentially leading to adverse responses in the surrounding brain. We designed and validated a murine model that mimics the hypoperfusion-reperfusion effect of bAVM and assessed BBB changes with dynamic contrast-enhanced magnetic resonance imaging (DCE-MRI).

**Objective:**

The goal is to explore perfusion alterations during bAVM progression and after surgical intervention, using DCE-MRI to track changes in real time.

**Methods:**

Cerebral hypoperfusion and venous congestion were induced in rats by bilateral external carotid artery ligation and unilateral jugular-carotid anastomosis. 21 days were allowed for chronic adaptative changes, then the fistula was closed by jugular ligation for reperfusion. Neurological examination and MRI were performed at 1, 7, and 21 days of evolution and 24 h after ligation. DCE findings was confirmed by IgG immunofluorescence.

**Results:**

MRI observations confirmed that the model successfully replicates arterial hypoperfusion and venous hypertension without signs of malignant oedema or ischemia. Neurofunctional assessment showed a progressive neurological decline with a tendency to improvement after shunt ligation. DCE-MRI showed progressive BBB disruption and restoration after ligation, objectively quantified by increases in Ktrans values. Similar results were obtained by immunofluorescence analysis.

**Conclusion:**

Our model provides a dynamic platform to investigate cerebral perfusion changes associated with bAVM progression and surgical resolution, offering novel insights into disease mechanisms and potential therapeutic strategies.

## Highlights

• Described murine model replicates bAVM hypoperfusion-reperfusion dynamics.
• DCE-MRI quantifies BBB disruption and recovery after fistula closure.
• BAVM model shows neurofunctional decline during onset and recovery after treatment.
• Ktrans values track BBB changes in bAVMs in real time.
• During the preparation of this work the author(s) don't used AI.

**Table:** Abbreviations

bAVM	Arteriovenous Malformation In The Brain
BBB	Blood-Brain Barrier
CCA	Common Carotid Artery
CJF	Carotid-Jugular Fistula
DCE-MRI	Dynamic Contrast-Enhanced Magnetic Resonance Imaging
DICOM	Digital Imaging and Communication In Medicine
DWI	Diffusion-Weighted
ECA	External Carotid Artery
ICA	Internal Carotid Artery
IF	Immunofluorescence
IJV	Internal Jugular Vein
NPPB	Normal Perfusion Pressure Breakthrough
O/N	Over/Night
PBS	Phosphate Buffer Saline
RT	Room Temperature
SSS	Superior Sagittal Sinus
TOF	Time Of Flight

## 1 Introduction

The presence of an arteriovenous malformation in the brain (bAVM) induces hemodynamic changes affecting the physiological reactions of intracranial vessels, which may influence the natural evolution and response to treatment (Leblanc et al., 2009), (Sekhon et al., 1997), (Rutledge et al., 2021), (Spetzler et al., 1978), (Lawton et al., 2015). The low-resistance arteriovenous shunt can induce vascular steal in surrounding territories and cause chronic hypoperfusion and loss of autoregulation (Hai et al., 2004), (Halim et al., 2004). Meanwhile, venous flow overload can induce venous hypertension and hinder the drainage of normal brain territories. Under these conditions, damage to the endothelium, loss of pericytes, proinflammatory states and, ultimately, blood-brain barrier (BBB) dysfunction may be responsible for bAVM manifestations such as hemorrhage, seizures and cognitive impairment (Hai et al., 2004), (Winkler et al., 2018), (M et al., 2017), (Daneman et al., 2010), (Sammons et al., 2011), (Keep et al., 2014). Conversely, bAVM treatment is thought to modify this longstanding hemodynamic condition abruptly, potentially leading to adverse reactions in the surrounding brain (Spetzler et al., 1978), (Gutiérrez-González et al., 2012), (Zacharia et al., 2012).

Therefore, concerns have been raised regarding the convenience of active treatment for bAVMs, particularly in non-ruptured cases (Mohr et al., 2014), (Pulli et al., 2019), (Rutledge et al., 2014), (Pedrosa et al., 2025). The very limited data available on the natural history of the disease makes it difficult to ponder benefits and interventional risks. In this sense, a longitudinal assessment of the neurovascular unit (i.e., BBB permeability), during bAVM evolution and after treatment, may help to understand how the brain and vasculature react to the presence of the shunt and its closedown (Chen et al., 2008), (Krithika and Sumi, 2020), (Ricciardelli et al., 2023). An interesting novelty in this regard is that these vascular changes can now be studied in vivo thanks to the evolution of neuroimaging techniques. Particularly, dynamic contrast-enhanced magnetic resonance imaging (DCE-MRI) has been proposed as a reliable tool to measure the permeability of BBB in several neuroinflammatory and neurovascular diseases (Varatharaj et al., 2019), (Heye et al., 2014), (Mosteiro et al., 2024).

Reproducing all of the above effects in a preclinical model of arteriovenous shunt may serve to test neuroprotective and vascular-modulating therapies. Previous animal models showed that creating an arteriovenous anastomosis in the cervical region was useful for studying the biology of arterialized veins, cerebral hypoperfusion and venous congestion effects (Raj and Stoodley, 2015). We build upon them to recreate the effects of treatment by closing the shunt after a latency period, thus inducing a reperfusion effect on the previously oligemic hemisphere. Our aim was to develop and validate the first arteriovenous shunt model able to explore the longitudinal changes in BBB permeability throughout the entire process (formation, evolution and resolution) by using DCE-MRI.

We hypothesize that our murine model effectively replicates the hypoperfusion-reperfusion dynamics observed in patients with bAVMs, as indicated by DCE-MRI. This model is expected to lead to measurable changes in blood-brain barrier (BBB) integrity. Additionally, we propose that surgical intervention to remove the shunt will restore BBB integrity, simulating the treatment of bAVM in human patients.

## 2 Materials and methods

### 2.1 Animals

This study was conducted according to Spanish Law (RD53/2013), in compliance with the EU Directive (2010/63/EU) for animal experimentation. Experiments were approved by the Institutional Ethics Committee (PNT 119/22) and carried out following the institutional animal care guidelines. Ethical license protocols and animal well-being were controlled periodically during the conduction of the project. The study is in accordance with ARRIVE guidelines.

Thirty-five male Wistar rats (Janvier), weighing 300–350g, were used in the study. Rats were housed in an animal room with strict humidity (50 %–60 %) and temperature (22–24 °C) control, under a 12-h day/night cycle. Groups of three animals were housed in plastic cages with free access to standard food and tap water.

### 2.2 Experimental groups

Fifteen animals were assigned to the hypoperfusion-reperfusion group, in which both arteriovenous fistula creation and subsequent ligation were performed. An additional group of 15 animals were assigned to the hypoperfusion-only group, in which the fistula was created but not treated. Finally, a set of 5 animals were assigned to the sham group, in which only bilateral ligation of both external carotid arteries was performed.

### 2.3 Surgical arteriovenous shunt creation

**Fig. 1:**
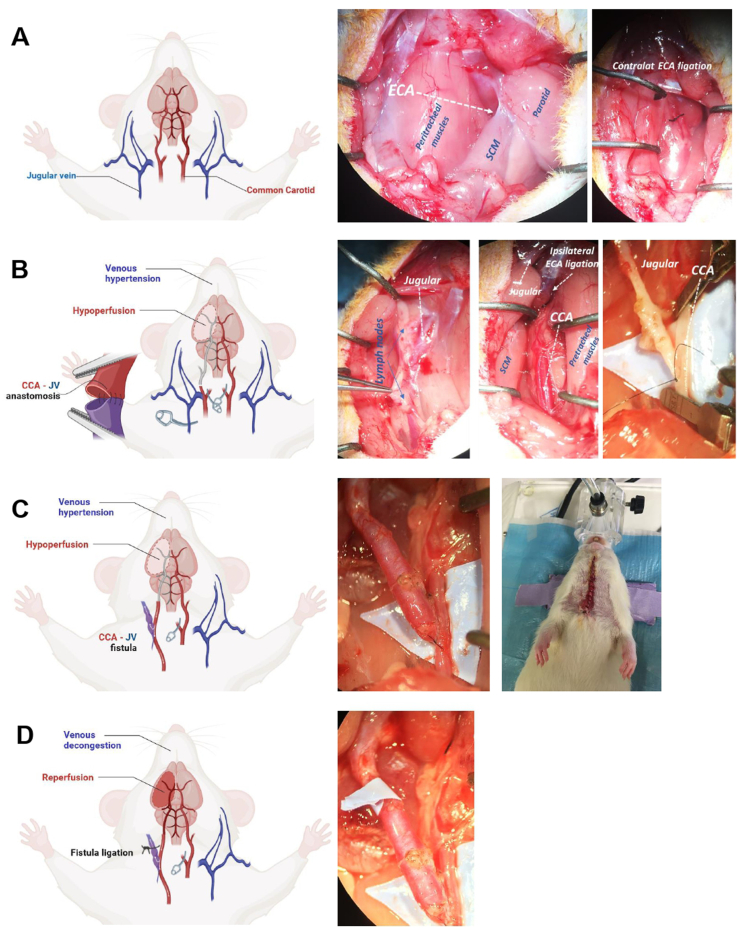
Illustration of the method for creating a carotid-jugular fistula (CJF). (A) The illustration shows the relevant vascular anatomy of the cervical region in the rat. The photography displays the intraoperative anatomy of the left (contralateral) cervical region. Dissection between the sternocleidomastoid muscle (SCM) and the peritracheal muscles leads to the vascular neck neurovascular bundle. The external carotid artery (ECA) is identified and ligated. (B) The illustration shows the series of vascular anastomosis and ligations that constitute the model. The ECA is bilaterally ligated. The right internal jugular vein (IJV) is ligated distally in the cervical area and the proximal graft is prepared for anastomosis with the common carotid artery (CCA). The photography shows the preparation of the jugular-carotid anastomosis. (C) The illustration shows the evolution of the jugular-carotid fistula, with the expected changes in the cerebral hemisphere, i.e., arterial hypoperfusion and venous congestion. The photography shows the patency of the jugular-carotid anastomosis, with an evident engorgement of the venous graft. On the right, the sutured cervical incision in the rat. (D) After 21 days of shunt evolution, the IJV is ligated distally to the anastomosis, while the CCA is maintained patent. The expected changes intracranially are arterial reperfusion.

Wistar rats were anaesthetized with 4 % inhaled isoflurane in an induction chamber and then maintained with 1–1.5 % isoflurane during the surgical procedure. Buprenorphine 0.5 mg/kg was administered subcutaneously before skin incision. An animal model of chronic cerebral hypoperfusion and venous congestion was created by a sequence of cervical vessel ligation and anastomosis, as shown in Fig. 1.

Animals were maintained under close postoperative surveillance for three days. Only buprenorphine (not anti-inflammatory drugs) was given every 12 h for pain relief. Physical examination was conducted daily, and corrective measures were applied to ensure animal well-being according to protocol. Animals were kept alive for 21 days to allow for chronic adaptative changes secondary to hypoperfusion to occur in the brain.

### 2.4 Arteriovenous shunt ligation (“bAVM treatment”)

Those animals assigned to the hypoperfusion-reperfusion group underwent a second surgery for arteriovenous anastomosis ligation. The second procedure was performed 21 days after the anastomosis creation.

Animals were again anaesthetized with 4 % isoflurane and maintained with a 1 % concentration. The cervical incision was reopened, and the carotid-jugular fistula (CJF) identified. To close the arteriovenous shunt and allow for hemispheric reperfusion, the jugular vein was ligated, and the common carotid (leading to the internal carotid) was kept patent (Fig. 1).

### 2.5 Neurological evaluations

Neurological functional testing was performed at days 1, 7, and 21 post-CJF creation using the Garcia Neuroscore (Garcia et al., 1995), ranging from 0 to 18 (Supplementary data). Animals were evaluated by a trained examiner in a blind fashion.

### 2.6 MRI evaluation of the CJF model and BBB changes

MRI was used at specific time points at 1, 7 and 21 days after CJF to evaluate the anastomosis patency and the ability of the model to reproduce the chronic hypoperfusion observed in human bAVMs, and to mimic the reperfusion phenomenon expected after bAVM resection (24h after shunt ligation). The protocol included sequences for anatomical, parenchymal, flow and BBB assessment, as detailed in Supplementary data. Data were processed with ROCKETSHIP software (Barnes et al., 2015).

The validation of the model was based on the neuroimaging confirmation of 1) The permeability of the arteriovenous anastomosis throughout the whole follow-up period; 2) the presence of signs of venous hypertension or flow overload intracranially; 3) the evidence of asymmetric intracranial arterial flow, as an indirect sign of hypoperfusion induced by the shunt; 4) the absence of structural damage (absence of irreversible ischemia).

The evaluation of the BBB permeability was done by DCE-MRI sequence. The region of interest was the whole cerebral hemisphere. Vascular input function was semiautomatically selected in the superior sagittal sinus and T1 mapping parametric fitting and DCE-MRI data were processed with ROCKETSHIP software (Barnes et al., 2015). The extended Tofts (Ex-tofts) pharmacokinetic model was used to obtain BBB permeability maps (Ktrans, expressed as mL/100 g/min units).

### 2.7 Histological evaluation: IgG immunofluorescence

Animals were euthanized by cardiac perfusion under anaesthesia 24h after the ligation of the shunt to allow for histological analysis of brain tissue. Immunofluorescence (IF) methods were applied to detect IgG and manually quantified under confocal microscopy. This served as a marker of BBB leakage. For more details of IF see Supplementary data.

### 2.8 Statistics

Rats were randomly allocated to shunt occlusion or non-occlusion groups. To minimize variability, all surgeries were performed by the same investigator. Subsequent histological and biochemical evaluations were performed blindly by other investigators. Groups were compared by analysis of variance and post hoc test or with *t*-test, as indicated in each figure legend. The parametric or non-parametric tests were selected after a Kolmogorov–Smirnov test or a Shapiro–Wilk test (for small-sized groups) for assessing normal distribution. GraphPad Prism 9 was used to run statistical analysis and graphic illustrations.

## 3 Results

### 3.1 Feasibility of the CJF model

In this study, animals were divided into three experimental groups to evaluate the effects of brain hypoperfusion and reperfusion. Fifteen animals were assigned to the hypoperfusion–reperfusion group, in which an arteriovenous anastomosis between the internal jugular vein and common carotid artery creation was created, and subsequently ligated after a latency period to allow for chronic vascular remodelling. Another group of fifteen animals were included in the hypoperfusion-only group, where the anastomosis was created but not ligated (“not treated”). Finally, a group of five animals was selected as control (sham) group, in which only bilateral ligation of both external carotid arteries was performed.

A total of 30 animals were subjected to CJF. Of these, 26 survived the 21-day protocol. Four animals died during the surgical procedure (Fig. 1), one was euthanized due to elevated distress according to supervision protocol, one experienced spontaneous death within 48 h post-surgery, and one died during MRI scanning at day 7 after surgery (Supplementary Fig. S1B).

Postoperative supervision scores (Supplementary Table S1) were high at 24–48 h (3–4), reflecting the degree of invasiveness of the procedure, with animals experiencing considerable difficulty in recovery during the first days. However, by 72 h, the supervision scores had notably decreased to a range of 0–2, suggesting a marked improvement in recovery (Supplementary Fig. S2). Despite bilateral external carotid artery (ECA) ligation, none of the animals developed skin necrosis on the head and neck.

### 3.2 Validation of the hypoperfusion-reperfusion CJF model with MRI

MRI assessment confirmed that the CJF remained patent throughout the whole follow-up period and that the model effectively reproduced the intended hemodynamic changes.

Time Of Flight (TOF) sequences provided valuable information on hemodynamic changes associated with the CJF model. The TOF sequence was used to verify the patency of the arteriovenous anastomosis over the following 21 days. Moreover, 3D-TOF reconstructions provided a comprehensive visualization of the vascular architecture. In all animals with a patent anastomosis, several changes were evident: (1) The absence of flow in both ECAs at the cervical level; (2) an asymmetric flow in the internal carotid artery (ICA) and right intracranial branches (middle cerebral artery) that showed restricted flow (narrowing) compared to the contralateral side; (3) an engorgement of the right internal jugular vein (IJV) from the point of the cervical anastomosis and retrogradely towards the intracranial compartment (Supplementary Fig. S3).

**Fig. 2:**
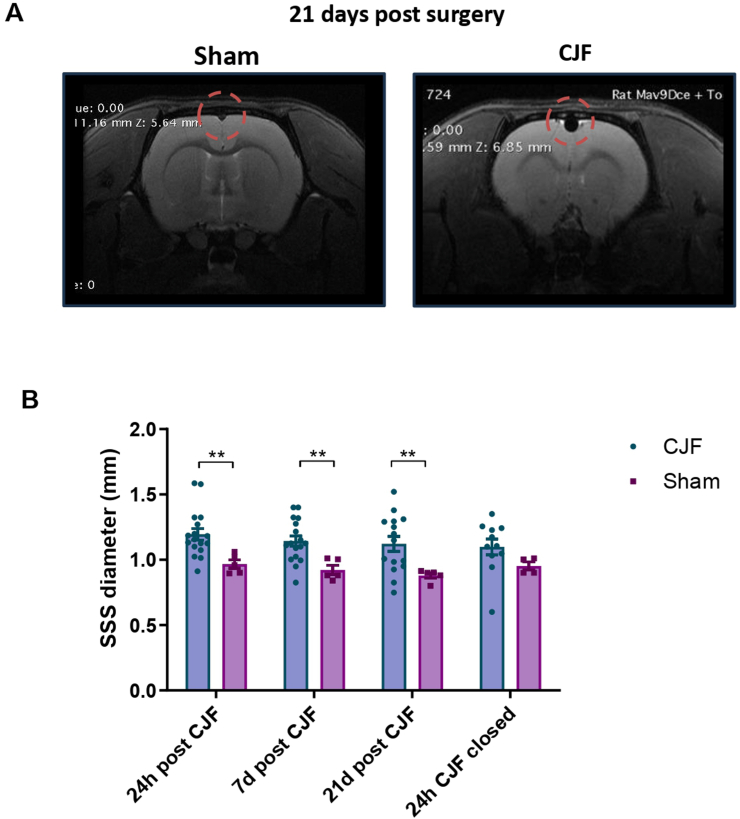
Superior Sagittal Sinus (SSS) dilation by MRI. (A) Diffusion-weighted imaging (DWI) reveals the SSS, highlighted by a red circle, in both the Sham (control, left) and CJF model at 21 days post-fistula creation. (B) Quantification of the maximum diameter of the SSS at three-time points: 24 h, 7 days, and 21 days following CJF creation, as well as 24 h after CJF closure. Measurements for the CJF model are shown in blue (n = 19) and for the sham group in purple (n = 5). Statistical analysis was performed using ANOVA; ∗∗ indicates p-value <0.01.

The impaired venous drainage and indirect signs of venous hypertension were further characterised by T2-weighted images, showing an enlargement of the superior sagittal sinus immediately (day+1) after CJF creation. Nonetheless, venous impairment did not induce venous infarction or malignant oedema (Fig. 2).

The asymmetry observed between the right and left ICA and their intracranial branches suggests a hypoperfusion state in the hemisphere ipsilateral to the CJF (Supplementary Fig. S3B). Furthermore, the absence of DWI lesions confirmed that the model recreates oligemia but not ischemia, as expected based on findings from human bAVMs (Fig. 2A). Overall, MRI results demonstrated that the model successfully replicates the hypoperfusion in cerebral parenchyma, along with venous hypertension typically associated with the presence of a bAVM.

An additional MRI evaluation was performed 24 h after closure of the arteriovenous shunt (IJV ligation with common carotid artery (CCA) and internal carotid artery (ICA) preservation). TOF confirmed the absence of flow through the shunt. We observed that the dilation of the veins persisted, but the TOF signal was less intense, which could be interpreted as an indirect sign of diminished flow at this level (Supplementary Figure S3 & Fig. 2B). This is probably due to the limited compliance of venous walls. No signs of hemorrhage or ischemia were observed.

Altogether, these MRI findings validate the CJF model as a reproducible method to induce and reverse cerebral hypoperfusion and venous hypertension.

### 3.3 BBB changes in the hypoperfusion-reperfusion model

Several dynamic changes were observed in BBB permeability following the creation of the CJF (hypoperfusion) and posterior ligation (reperfusion). These changes were studied in vivo with DCE-MRI and subsequently confirmed by IF analysis of tissue samples.

**Fig. 3:**
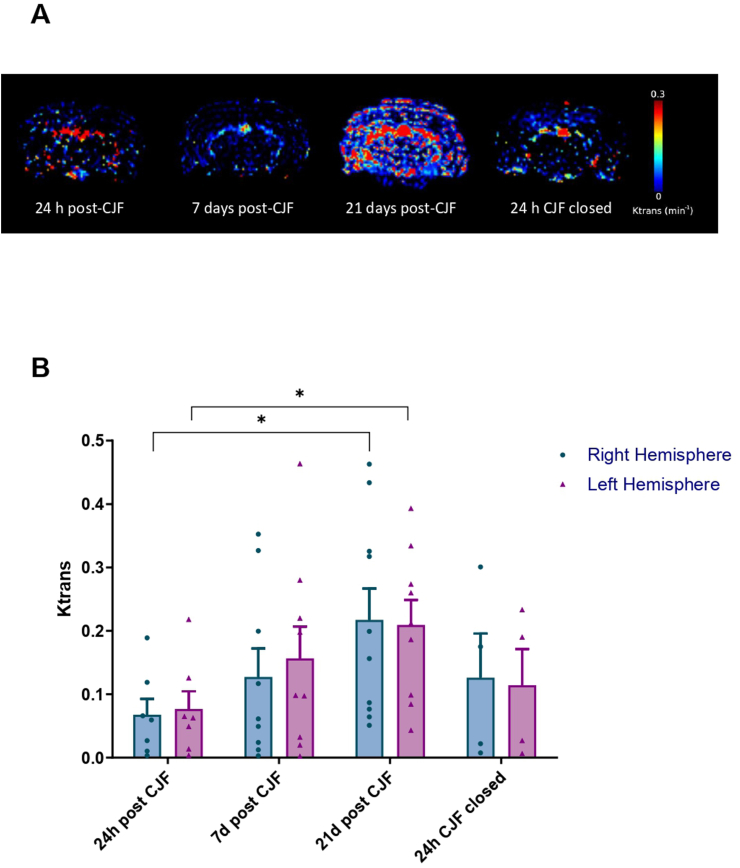
Evolution of BBB permeability, assessed with DCE-MRI before and after carotid-jugular fistula (CJF) closure. (A) Ktrans maps illustrating BBB permeability at three time points (24 h, 7 days, and 21 days) following CJF creation, and 24 h post-CJF closure in a representative CJF-rat model. Areas with red indicate elevated Ktrans values, signifying increased BBB disruption, while blue regions denote low Ktrans values, reflecting minimal BBB disruption. (B) Quantitative analysis of Ktrans in the right (blue) and left (purple) hemispheres of the CJF-rat model at the specified time points: 24 h, 7 days, and 21 days post-CJF creation, along with 24 h after CJF closure. Statistical significance was determined using ANOVA; ∗ indicates p-value <0.05.

DCE-MRI showed a progressive disruption of BBB throughout the 21 days after CJF generation. The permeability constant (Ktrans) increased from day 1 (0.072 ± 0.070) to 21 (0.208 ± 0.135, p = 0.031). Importantly, this increase in Ktrans was followed by a marked decrease after fistula ligation (0.0208 ± 0.135 vs 0.120 ± 0.125, p = 0.293); however, Ktrans levels did not return to baseline at 24 h (Fig. 3).

**Fig. 4:**
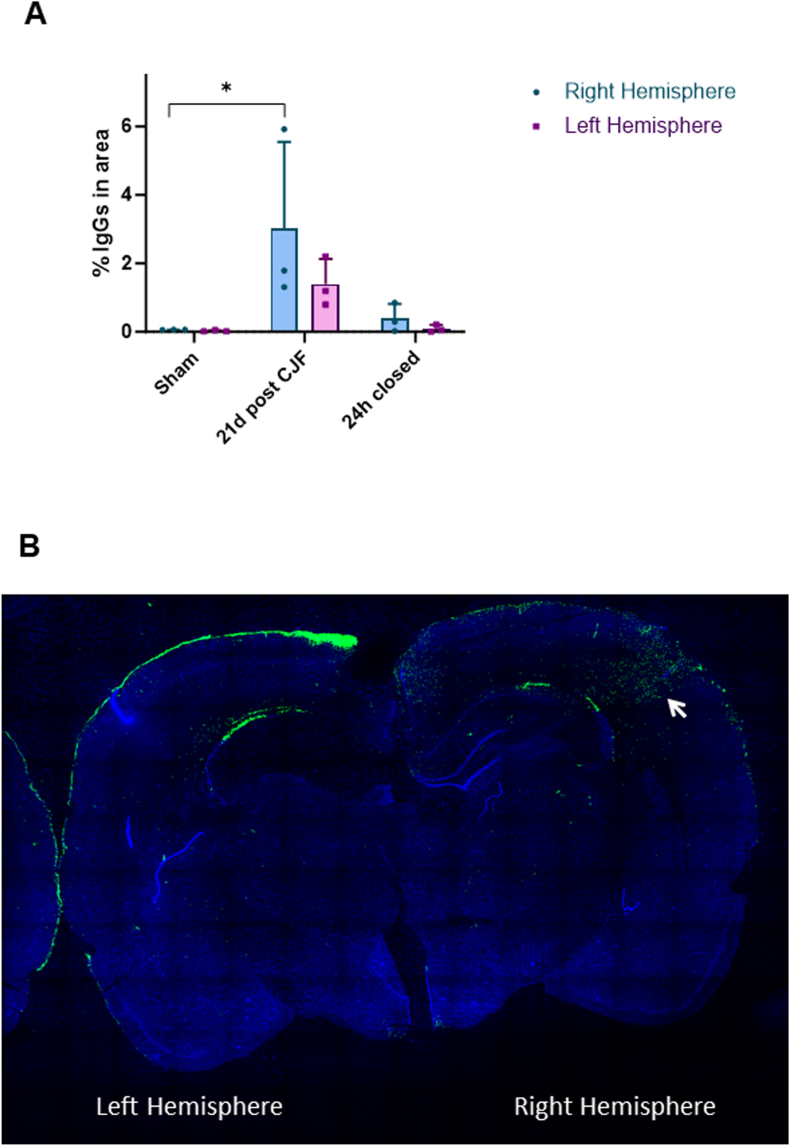
BBB permeability analyzed by IF. (A) Quantification of immunoglobulin G (IgG) levels in the right (blue) and left (purple) hemispheres of sham (control), as well as at 21 days post-carotid-jugular fistula (CJF) creation and 24 h after CJF closure of CJF-rat model. Statistical analysis was performed using ANOVA; ∗ indicates p < 0.05. (B) Immunofluorescence image depicting anti-IgG staining 21 days after CJF creation. The arrow indicates the presence of IgG fluorescence. IgGs are shown in green, while nuclei are counterstained in blue with DAPI.

Similar results were then obtained by immunofluorescence (IF) analysis of IgGs. In the hypoperfusion-only group, IgG levels in brain parenchyma were higher after 21 days of CJF creation, compared to the sham control group (0.049 ± 0.008 vs 2.152 ± 1.514, p = 0.038). IgGs were more prominent in the cortex of the right hemisphere. In the hypoperfusion-reperfusion group, IgG levels significantly decreased after CJF ligation (2.152 ± 1.514 vs 0.246 ± 0.196, p = 0.044) (Fig. 4).

Together, DCE-MRI and IF analyses demonstrated that BBB permeability dynamically changes following the creation and subsequent closure of the arteriovenous shunt, indicating that this model effectively captures both the progression and partial reversibility of BBB disruption associated with bAVM-like hemodynamic alterations.

### 3.4 Neurological performance

The neurofunctional evaluation revealed a pattern of progressive neurological decline over time of the arteriovenous shunt (Supplementary Fig. S4). Interestingly, 24 h after fistula ligation, a trend toward neurological recovery was observed, although this improvement did not reach statistical significance. These findings suggest that the hemodynamic changes induced by the CJF model are functionally relevant and, at least in part, reversible.

## 4 Discussion

In this original investigation, we provide a well-founded murine model of the hemodynamic changes seen in the brain before and after curative treatment of arteriovenous shunts (such as bAVMs), namely the hypoperfusion-reperfusion biphasic state. This is the first study to use MRI to longitudinally track long-standing cerebrovascular changes after the formation of an arteriovenous shunt in an in vivo model, and to observe the changes after the surgical treatment of the shunt. With this model, we were able to simulate the blood-brain barrier disruption and recovery observed in bAVM patients before and after curative treatment (Mosteiro et al., 2024).

The model consists of a carotid-jugular fistula plus bilateral ECA ligation, and the validity of the design was supported by serial evaluations with cervical and cerebral MRI up to day 21. Treatment of the arteriovenous shunt (“bAVM”) is simulated by jugular vein ligation and preservation of the carotid flow and further evaluated with MRI. Ultimately, we applied the model to study BBB alterations in the brain hereby exposed to chronic hypoperfusion and sudden reperfusion, mimicking the flow behaviour of a bAVM and its subsequent treatment.

One of the key contributions of this study is the novelty of applying MRI to monitor the arteriovenous shunt model and to address dynamic changes in BBB during the evolution of the shunt and after treatment. Remarkably, in a translational comparison, our experimental model seemed to reproduce the changes observed in a cohort of patients treated for bAVM (Mosteiro et al., 2024). This unprecedented experience may provide novel insight into the physiopathology of bAVMs, the changes induced in the surrounding brain, the mechanisms underlying symptoms, and the brain adaptative responses after curative treatment.

### 4.1 Development of bAVM animal models for hemodynamic investigation

Hemodynamic models of bAVMs gained interest in understanding how the surrounding brain parenchyma reacts to the restoration of normal arterial flow after the curative excision of the arteriovenous shunt. In fact, Spetzler et al. designed a rat model of CJF to demonstrate their theory of normal perfusion pressure breakthrough (NPPB) (Spetzler et al., 1978). This model, which involved the anastomosis of the CCA to the jugular vein, effectively created non-infarcted cerebral hypoperfusion to simulate conditions surrounding high-flow bAVMs. Their findings indicated that chronic oligemic brain tissue in proximity to such lesions experienced a diminished cerebrovascular autoregulation capacity. However, subsequent investigations suggested the changes induced by the CJF model were minimal and transient, calling into question the model's efficacy in elucidating the NPPB phenomenon fully (Sakaki et al., 1992), (Tokiwa et al., 1995).

To address these limitations, Morgan and colleagues introduced a modified CJF model in rats, allowing for a more controlled examination of hemodynamic changes (Morgan et al., 1989). Later, Hai et al. further refined the murine model, providing evidence linking the NPPB mechanism to acute ischemia and reperfusion injury (Hai et al., 2004). They demonstrated significant alterations in cerebral blood flow following the ligation of the fistula, resulting in BBB disruption under hypertensive conditions. Meanwhile, the concept of "occlusive hyperemia" was proposed as an alternative to explain the brain oedema and hemorrhage associated with bAVM resections, acknowledging the importance of the venous side of the bAVM circuit. Bederson et al. echoed this theory by demonstrating in a rat model that venous occlusion could lead to severe complications (Bederson et al., 1991). A summary of the main experimental models developed to study the hemodynamic mechanisms related to bAVMs is presented in Table 1.

**Table 1:** Summary of experimental animal models developed to study the hemodynamic mechanisms associated with brain arteriovenous malformations (bAVMs). The table lists the main characteristics, species used, benefits, and drawbacks of each model described in the literature.

Model	Citation	Animal	Benefits	Drawbacks
CJF model (carotid-jugular fistula	Spetzler et al.	Rat	Simulates a high-flow environment similar to bAVMs, which provides an experimental basis to study impaired cerebrovascular autoregulation	Hemodynamic changes are minimal and transient and does not fully reproduce the Normal Perfusion Pressure Breakthrough (NPPB) phenomenon.
	Morgan et al.	Rat	Offers better control over hemodynamic conditions; enables systematic study of local flow effects.	Technically more complex; still fails to fully replicate the human bAVM pathophysiology.
	Hai et al.	Mice	Allows investigation of ischemia–reperfusion injury and blood–brain barrier (BBB) disruption; provides a more translational model.	Hypertensive response can be difficult to regulate; may not reflect the chronic high-flow state of bAVMs.
Venous occlusion model (“occlusive hyperemia”)	Bederson et al.	Rat	Highlights the role of the venous component in post-resection complications; helps explain oedema and hemorrhage mechanisms.	Does not reproduce the arterial high-flow aspect of bAVMs; may induce severe brain injury that complicates interpretation.

Our model builds upon previous ones by adding certain modifications in both the arterial and venous sides. Compared to Morgan's, in which the ICA was ligated proximally and then anastomosed to the jugular vein, we preserve the ICA during the arteriovenous anastomosis, which enables us to then mimic the “treatment" of the malformation by ligating the jugular while maintaining the ICA flow. Compared to Bederson's, which is intended to increase venous congestion, we do not ligate both jugular veins (i.e., also the contralateral). In fact, we maintain both patent during the 21 days of arteriovenous evolution and increase the venous load by 1) performing an arteriovenous anastomosis with the ipsilateral CCA, and 2) ligating both ECA to increase arterial flow in the CCA and, consequently, in the jugular vein. Then, when we performed the "treatment”, we only ligated the ipsilateral jugular vein, allowing the brain to drain through the contralateral side.

With these premises, we were able to demonstrate the progressive development of arterial hypoperfusion and venous congestion as we followed changes induced by the arteriovenous shunt with MRI.

Although the carotid–jugular fistula (CJF) model does not reproduce a true cerebral arteriovenous malformation (bAVM), it offers important experimental advantages for investigating the hemodynamic and functional consequences of arteriovenous shunting. Compared with genetic models, which can produce multiple AVMs of variable size and location, the CJF model allows for a highly controlled and reproducible induction of an arteriovenous shunt, enabling consistent assessment of cerebral hemodynamic and (BBB function. Moreover, this model uniquely permits longitudinal evaluation before and after shunt closure, providing valuable insights into the dynamic recovery of cerebral perfusion and BBB permeability following the normalization of flow. Although the shunt itself is extracerebral, the effects have a direct impact on the brain. And our MRI analyses (including DCE-MRI and TOF imaging) confirm that the CJF model induces measurable alterations in cerebral circulation, supporting its use as a reliable tool to study AVM-related pathophysiology and therapeutic interventions.

### 4.2 Development of bAVM animal models for BBB investigation

Lately, hemodynamic factors associated with bAVMs have been linked to wall shear stress and vessel wall inflammation. The inflammatory theory of bAVM has gained acceptance as a plausible explanation for the development of symptoms and eventual rupture. Although several laboratory investigations have supported this idea, the application of an animal model based on the flow-dynamics of bAVMs to study BBB disturbances and neuroinflammation has not yet been reported.

A major challenge for this task is selecting a tool that allows the assessment and quantification of the BBB permeability in vivo, preferably in a non-invasive manner that could be repeated at different time points. By applying DCE-MRI to our hemodynamic model of an arteriovenous shunt, we were able to demonstrate the dynamic changes in BBB permeability, in parallel with disease progression and curative treatment of the malformation. These image-based fluctuations were confirmed histologically by IF analysis of IgG. The capability to follow the same animal across different time points is notably advantageous, as it reduces inter-individual variability that often complicates the interpretation of pharmacological studies. This model provides a unique opportunity to assess the temporal dynamics of BBB restoration and could be used to test neuroprotective agents.

This approach may serve as a useful preclinical platform to test therapeutic interventions aimed at protecting or restoring the BBB and to explore mechanisms of brain recovery after bAVM treatment.

### 4.3 BBB alterations in bAVMs: a translational research narrative

Ultimately, to be useful in translational research, experimental models should be able to reproduce the biological responses observed in humans, at least at some level. In this regard, the greatest strength of our murine model is that it was able to reproduce the dynamic changes observed in BBB permeability in a cohort of patients treated for bAVM (Mosteiro et al., 2024). Remarkably, the changes in K_trans_ observed in the parenchyma surrounding the bAVM in patients, before and after surgical resection±endovascular embolization, are consistent with those observed in the rat cerebral hemisphere after the creation and ligation of the cervical CJF.

A limitation of this study is that, although the model replicates the hemodynamic and BBB changes associated with bAVMs, it does not reproduce the structural and genetic complexity of true bAVMs. Nevertheless, this controlled setting enables the isolation of hemodynamic variables that would be difficult to assess in clinical studies.

## 5 Limitations

This study showed several limitations. The first one is conceptual, as the cervical arterio-venous anastomosis induces haemodynamic changes globally in the cerebral hemisphere of the rat, which contrasts with the loco-regional changes seen in the perimalformative brain surrounding the bAVM in humans. In this sense, it may be argued that the model is actually replicating the effect of high-grade (Spetzler-Martin IV-V) lesions, rather than smaller shunts. Second, the loss of subjects at specific times during the MRI evaluation due to the presence of physiological stress signals that prevented them from undergo sedation or technical problems during venous canulation for contrast administration. Third, limitations inherent to the neurological functional test performed (Garcia Neuroscore), which may not reflect all the changes in complexity seen in the human cognitive function during bAVM development. Finally, animals were assessed at a single time point after "curative treatment” (24h); a longer follow-up period could have provided more comprehensive information on the long-lasting effects of the intervention.

## 6 Conclusions

Our study introduces a robust murine model that captures the hemodynamic alterations in the brain both before and after the surgical removal of an arteriovenous shunt. The results of this research offer valuable new perspectives on the underlying physiopathology of bAVMs, the impact these arteriovenous shunts have on surrounding brain tissue, the processes contributing to symptom development, and the mechanisms of brain adaptation following surgical intervention. These insights may enhance our understanding of bAVMs and improve approaches to their treatment.

## Informed consent statement

Not applicable.

## Author contributions

L.P., A.M., A.M., A.R-H, and R.T. contribute to conceptualization; L.P., A.M., L.A., A.M.M., A.R-H., and R.T. contribute to the methodology; L.P. and A.M. analyzed the results; L.P., A.M., M.C., G.C., L.A., A.P., S.A., A.M.M., A.R-H., and R.T. conceived the experiments and investigation; L.P. and A.M. conducted the experiments; L.P., A.M., and R.T. contribute to the writing—original draft; L.P., A.M., and R.T. contribute to the visualization of article; A.M.M., A.R-H., and R.T. supervised the study. All authors reviewed the manuscript.

## Institutional review board statement

This study was conducted according to Spanish Law (RD53/2013), in compliance with the EU Directive (2010/63/EU) for animal experimentation. All experiments involving animals were approved by the Institutional Ethics Committee (PNT 119/22) and carried out following the institutional animal care guidelines. Ethical license protocols and animal well-being were controlled periodically during the conduction of experiments.

## Data availability statement

All data generated or analyzed during this study are included in this published article and its supplementary information files.

## Funding

This research was funded by the competitive grant to Ramon Torné and Ana Rodríguez-Hernández of Fundació la Marató TV3 (248/C/2020). This research is also founded by ISCIII PI22/01290 and ISCIII PI22/01052.

## Conflicts of interest

The authors declare no conflicts of interest.

## Supplementary Materials:

The following supporting information can be downloaded at: https://www.mdpi.com/article/doi/s1, Supplementary Figure S1: Overview of experimental design; Supplementary Figure S2: Supervision score results after CJF creation; Supplementary Figure S3: Patency of the arteriovenous fistula throughout the 21 days; Supplementary Figure S4: Neuroscore results evaluated by the Garcia test.
