# Supplementary material for: Blood-brain barrier dysfunction in cerebral arteriovenous malformations. A murine model of hypoperfusion-reperfusion injury assessed with dynamic contrast-enhanced MRI
Source: Brain Spine. 2025 Nov 10;5:105871. doi: 10.1016/j.bas.2025.105871 (PMC12666813; doi:10.1016/j.bas.2025.105871)
Supplement: Multimedia component 1 [file mmc1.docx]

**Supplementary methods**

**Neurological Evaluations**

Neurological functional testing was performed on days 1, 7, 14 and 21 post-CJF creation using the Garcia Neuroscore. Animals were evaluated by a trained examiner in a blind fashion. The Garcia Neuroscore consists of 6 subtests: spontaneous activity, body proprioception, vibrissae touch, limb symmetry, forelimb outstretching and climbing.

For spontaneous activity, the animal instinctive activity in a novel environment (cage) was observed for 5 minutes. For body proprioception, the animal’s trunk was stimulated using a cotton swap and the motor reaction was noted. For vibrissae touch, a cotton swab was used to gently touch vibrissae back to front; again, the motor response was noted. For limb symmetry, the animal was suspended by the tail to assess the bilateral movement of the forelimbs. For forelimb outstretching, the animal was advanced forward through a flat surface up to the edge of the table, and the reflex response of the forelimbs was noted. For climbing, the animal was placed on a gripping surface (20×42 cm) aligned in a 45° angle and elevated 36 cm from the surface of a table, and its voluntary climbing movements were observed. The total score for the Garcia Neuroscore ranges from 0 (maximum neurological impairment) to 18 (no neurological deficits).

**TOF sequence processing**

DICOM images obtained from TOF-MRI sequences were exported for reconstruction and used to create 3D maps of the vessel flow to facilitate the interpretation of the vascular patterns. The initial DICOM files were imported into ImageJ, where they were organized into image stacks corresponding to the three-dimensional volume of interest. where they were organized into image stacks corresponding to the three-dimensional volume of interest. Subsequently, we applied a Z projection to the image stack to generate a two-dimensional representation of the volumetric data. Specifically, we utilized the "max intensity" projection type, which highlights the most intense values along the Z-axis, thereby enhancing relevant anatomical features and improving visualization for subsequent assessment. This method allows for a clearer interpretation of the structures captured in the TOF MRI images.

## **Histological evaluation: IgG Immunofluorescence**

For histological analysis, animals were anesthetised with 4% isoflurane and transcardially perfused with 100 ml of phosphate buffer saline (PBS) 0.1 mol/L, pH 7.4, containing 100IU unfractionated heparin, followed by 50 ml of chilled paraformaldehyde (4%) in PBS. The brains were carefully removed from the skull and post-fixed O/N at 4°C, then transferred to 30% sucrose in 0.1 mol/L phosphate buffer for 48h until equilibration. Coronal brain 50 μm-thick fresh sections were cut serially with automated vibrating blade microtome (LEICA VT1200S vibratome).

Tissue sections were incubated in wells containing a blocking solution of 3% donkey serum in PBS and a dilution of 1:500 anti-IgG antibody (711-065-152, Lot. Nr. 131559, Jackson ImmunoResearch, Ely, United Kingdom, dilution 1:500) for 1.5 hours at room temperature (RT). After antibody incubation, the sections were washed thoroughly with PBS and subsequently stained with DAPI for 15 minutes to visualize nuclei. Once the staining procedure was complete, the sections were mounted using an Fluoromount™ Aqueous Mounting Medium (Sigma, F4680) and allowed to dry for 2 hours. The prepared slides were then stored at 4°C until image acquisition via microscopy.

Confocal microscopy assessment was done using a sequential scanning mode to avoid bleed-through effects with a Leica TCS SP8 confocal microscope. Light excitation at 488 nm for IgG signals was used. Large view images were acquired by a 10x objective and automatically stitched with 10% overlap. Large view images served as a reference to identify the cortical regions of interest for subsequent analysis.

Digital image analysis was done using ImageJ. Briefly, each image was made bidimensional by max intensity projection and channels separated (blue for DAPI and green for IgGs). Following the creation of the 2D images, a threshold value to distinguish the positive IgG signal from the background was applied. This threshold was determined based on the negative-control sample, which did not undergo incubation with the IgG antibodies, ensuring that the selection criteria effectively minimized false positives.

For the area of analysis, we manually defined the region of interest (ROI) by selecting the entire brain section and both hemispheres. Within this predetermined area, we calculated the positivity of IgGs by quantifying the green channel signal, thereby assessing the immunoreactivity of the tissue sections.

## **MRI evaluation of the CJF model**

MRI was used at specific time points (days +1, +7, +21) to evaluate the fistula patency and the ability of the model to reproduce the chronic hypoperfusion seen in the perimalformative area of human bAVMs, and to mimic the reperfusion phenomenon observed in this area after bAVM resection (24h after ligation). The protocol included sequences for anatomical, parenchymal, flow and BBB assessment, as detailed in Supplementary data.

7 Tesla MRI was performed under sedation (1-1.5% isoflurane) at day 1, 7 and 21 after CJF creation, and 24 hours after ligation. The protocol included T1-weighted, T2-weighted, Diffusion-weighted (DWI, b=0,1000 s/mm2, TE1-TE2/TR 52-87/5600 ms, FA 180º, FOV 220x220mm, matrix 160x160, 40 slices, thickness 3mm) and Time of Flight (TOF, TE/TR 3.50/21 ms, FA 18º, FOV 200x200mm, matrix 384x384, 36 slices, thickness 0.52mm). The examination included both the head and the neck of the rat.

Maximum diameter of superior sagittal sinus (SSS) was measured with ITKSnap v.3.8 software (Cognitica, Philadelphia, USA). TOF-MRI sequences were reconstructed into 3D vessel-maps with ImageJ to facilitate the visualization of vascular flow.

**DCE-MRI evaluation of BBB permeability**

The integrity of the BBB was investigated by DCE-MRI at the same time-points (**Supplementary Figure S1A**). The DCE-MRI acquisition (TE/TR 1.73/4.87 ms, FOV 240x180, matrix 192x144, 44 slices, thickness 3 mm, 60 volumes, total acquisition time 5min24s) included a variable flip angle T1 mapping sequence (with same parameters and resolution as DCE-MRI and 8 different FA:1,3,5,7,9,11,13 and 15º). Five volumes of the DCE-MRI were acquired before the injection of Gadolinium (MultiHance^®^, Bracco, Italy, #0270-5164-14) at a dose of 1.5 mL/kg.

Vascular input function was semiautomatically selected in the superior sagittal sinus and T1 mapping parametric fitting and DCE-MRI data were processed with ROCKETSHIP software^20^. Extended TOFs pharmacokinetic model was used to obtain BBB permeability maps (Ktrans, mL/100g/min units).

**Supplementary Tables**

Supplementary Table S1: Supervision protocol for postoperative monitoring

| **Parameter** | **Observation / Condition** | **Score** |
| --- | --- | --- |
| **1. Weight loss** | No weight loss or normal growth | 0 |
|  | Weight loss < 10% | 1 |
|  | Weight loss 10–20%; possible alteration in stool appearance or amount | 2 |
|  | Weight loss > 20%; animal does not consume food or water | 5 |
| **2. Appearance** | Normal appearance | 0 |
|  | Poor coat condition (ruffled, dull, and/or dirty fur) | 1 |
|  | One or more of the following: mucous and/or bloody secretions from any orifice, diarrhea, palpable enlarged organs (lymph nodes, spleen, liver) | 3 |
|  | One or more of the following: abdominal distension of any origin, dyspnea (particularly if accompanied by nasal discharge and/or cyanosis), cachexia | 5 |
| **3. Unprovoked behavior** | Normal behavior | 0 |
|  | Slight difficulty moving normally | 1 |
|  | Difficulty reaching food or water, isolation from cage mates, decreased movement, abnormal posture, animal excessively restless or immobile | 3 |
|  | Self-mutilation or abnormal vocalizations | 5 |
| **4. Response to handling** | Normal behavior | 0 |
|  | Mild changes: tense, apathetic, or nervous during handling | 1 |
|  | Moderate changes: very anxious during handling (e.g., tremors) | 3 |
|  | Aggressive or comatose animal | 5 |
| **5. Wounds** | No wounds observed | 0 |
|  | Minor or moderate scratches, bites, or dermatitis | 1 |
|  | Non-healing wounds, severe aggression injuries, or severe/chronic dermatitis | 3 |

**Supplementary Figures**


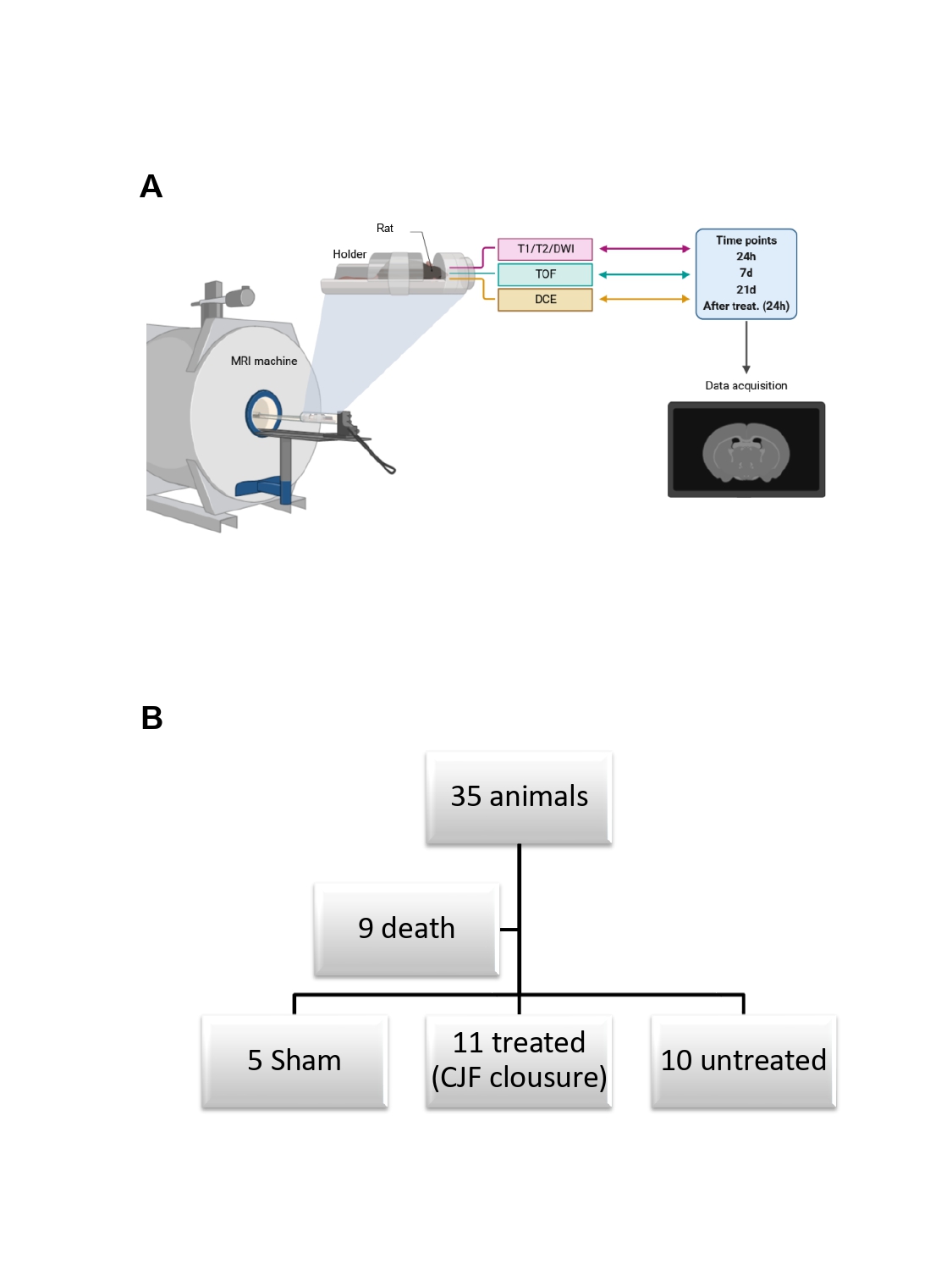


**Supplementary Figure S1:** **Overview of** **experimental design**. (A) Schematic representation of the MRI experimental timeline. T1, T2, Time-of-Flight (TOF), and Dynamic Contrast-Enhanced MRI (DCE-MRI) were conducted at 24 hours, 7 days, and 21 days post-CJF creation, as well as 24 hours following CJF ligation. (B) Summary of animal groups. Out of the initial 35 subjects, 9 animals succumbed during the study. Of the remaining, 11 underwent CJF ligation at 21 days, while 10 animals maintained a permeable fistula throughout the protocol.


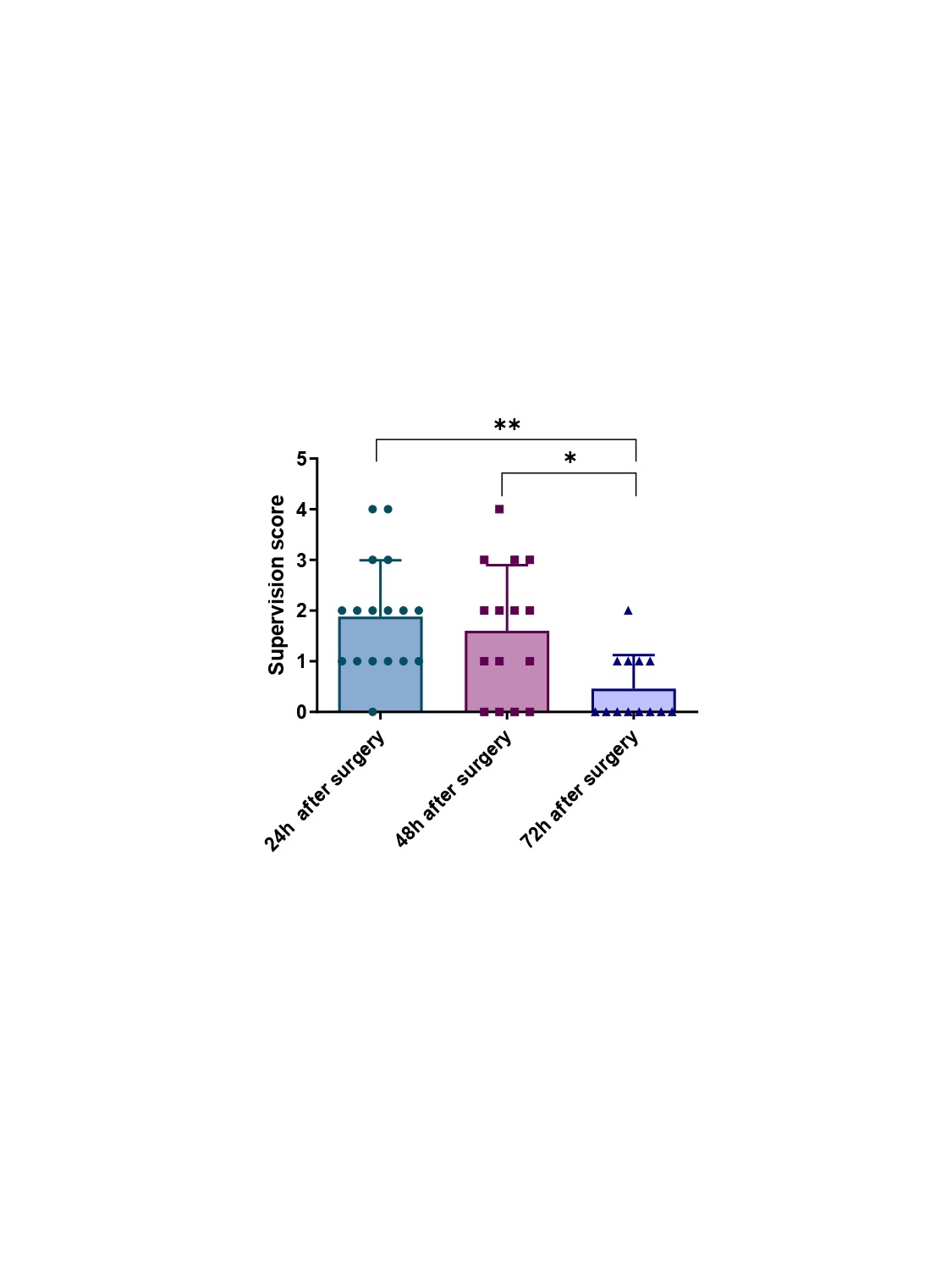


**Supplementary Figure S2:** Supervision score results after CJF creation. The results indicate a significant decrease in supervision scores at both 48- and 72-hours post-surgery, as determined by two-way ANOVA (p < 0.05).

**Supplementary Figure S3: Patency of the arteriovenous fistula throughout the 21 days.** Time-of-flight (TOF) images from a representative carotid-jugular fistula (CJF) model (B) and sham control (A) at 24 hours, 7 days, and 21 days post-CJF creation, as well as 24 hours after the closure of the CJF. The common carotid artery has been indicated with an orange arrow, the internal carotid with a red arrow, the internal jugular vein with a blue arrow, and the carotid-jugular fistula with a purple arrow.


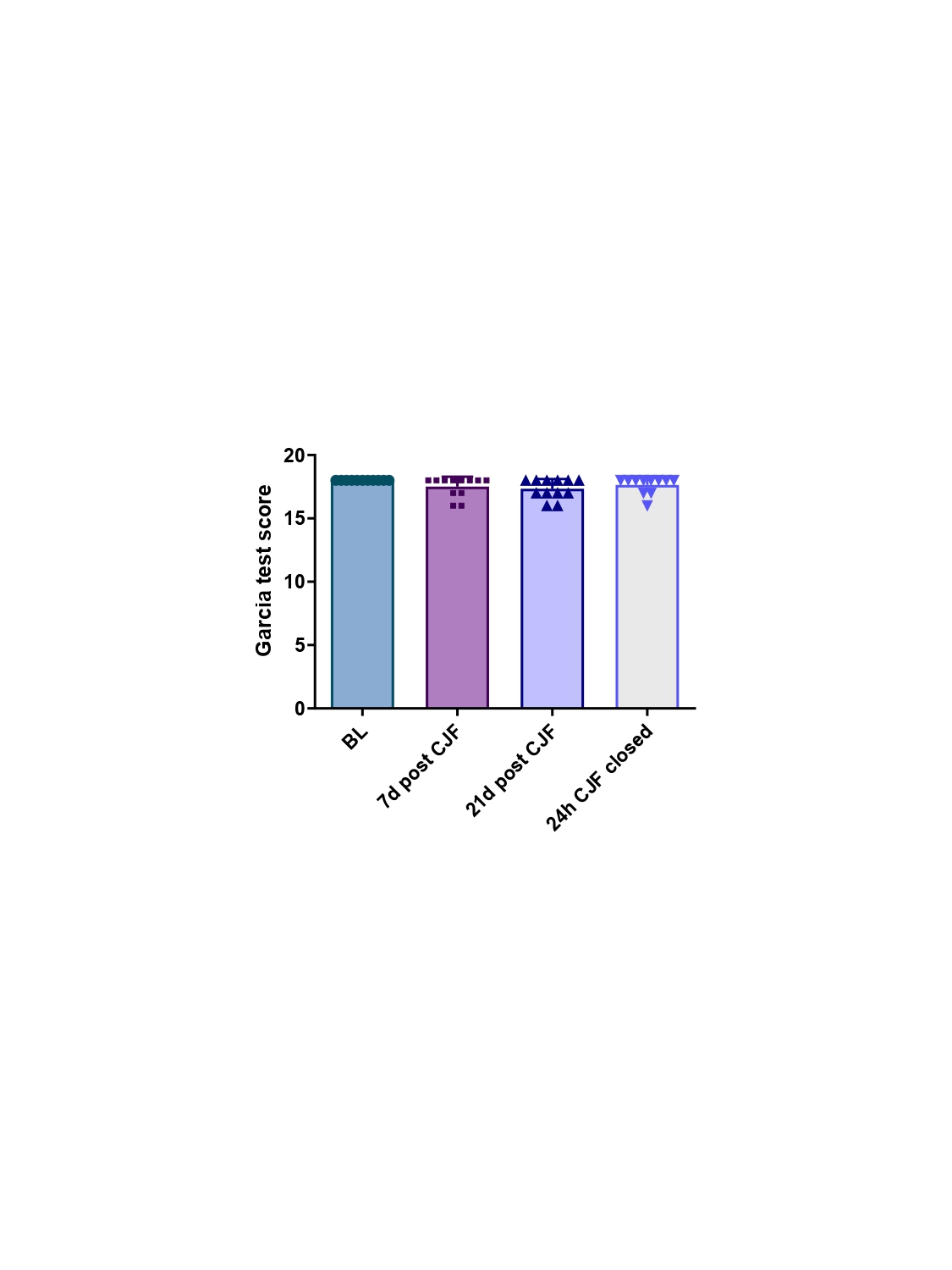


**Supplementary Figure S4: Neuroscore results evaluated by the Garcia test.** The neuroscore showed a trend toward the worst outcome after CJF creation and improved after CJF closure (two‐way ANOVA, p = 0.052).
